# A rare case of Dyke-Davidoff-Masson syndrome with concurrent metabolic syndrome

**DOI:** 10.1016/j.radcr.2025.06.017

**Published:** 2025-07-08

**Authors:** Sushmitha Rameshbabu, Naveenkumar Nallathambi, D Shaaruk, Imaduddin Mohammed, Neha Syed

**Affiliations:** a Institute of Internal Medicine, Madras Medical College, Chennai, India; b Junior Resident, Royal care Hospital, Coimbatore, India; c Department of Medicine, Shadan Institute of Medical Sciences, Hyderabad, India; d Department of Medicine, Salem Medical College, Tamil Nadu, India

**Keywords:** Cerebral hemiatrophy, Early-onset epilepsy, Hemiplegia, Dyke-Davidoff-Masson syndrome, Metabolic syndrome, Case report

## Abstract

Dyke-Davidoff-Masson syndrome (DDMS) is a rare neurological condition resulting from prenatal or early childhood brain injury, characterized by seizures, hemiparesis, facial asymmetry, and intellectual disability, with imaging findings including cerebral hemiatrophy, calvarial thickening, and mastoid cell hyperpneumatization. We present the case of a 13-year-old male who exhibited recurrent convulsive seizures predominantly affecting his left side. MRI revealed right-sided cerebral hemiatrophy with ipsilateral calvarial thickening and mastoid hyperpneumatization, consistent with DDMS. Notably, this case was further complicated by coexisting metabolic syndrome, a rare comorbidity in DDMS, which increased the complexity of diagnosis and management. This case highlights the importance of considering DDMS in pediatric patients presenting with focal seizures and hemiparesis, and emphasizes the need to recognize atypical clinical associations such as metabolic syndrome. Early diagnosis and a multidisciplinary approach are essential to improve outcomes and guide future research into broader manifestations of DDMS.

## Introduction

Dyke-Davidoff-Masson syndrome (DDMS) is a rare neurological disorder that results from brain injury sustained during fetal development or early childhood [1]. The syndrome presents with a spectrum of clinical features, including facial asymmetry, recurrent seizures, contralateral hemiparesis, learning disabilities, and intellectual impairments [2]. Radiological findings typically reveal unilateral cerebral atrophy with compensatory hypertrophy of the skull and sinuses on the affected side, which aids in diagnosis [3]. Given the limited number of documented cases and the potential for misdiagnosis, DDMS can have a profound impact on patient development and quality of life [2]. Since its initial description in 1933, fewer than 100 cases have been reported in the medical literature, with the majority occurring in children and only 21 cases documented in adults [4]. Here, we report a case of a 13-year-old male with a history of seizures on antiepileptic therapy who presented with sudden-onset involuntary limb movements. The concurrent presence of metabolic syndrome in this patient further complicates the clinical picture, highlights the need for comprehensive and multidisciplinary management strategies.

## Case presentation

A 13-year-old male presented to the outpatient department with complaints of rhythmic, jerky, involuntary movements of the left upper extremity. These episodes were sudden in onset, occurred approximately 10 times per day, and each lasted for 15-20 minutes. Importantly, the movements were not associated with loss of consciousness, tongue biting, frothing, or involuntary urination/defecation. There was no postictal confusion or drowsiness reported. These symptoms had been present for 1 day before presentation.

The patient had a past medical history significant for epileptic seizures starting at the age of 8 years, initially presenting with an episode of generalized tonic-clonic seizures followed by progressive weakness of the left side of the body. Facial deviation towards the right side was also noted at that time. There had been a progressive, irreversible left-sided hemiparesis since the initial episode. The patient had been started on antiepileptic therapy but had poor adherence to medications, with intermittent discontinuation due to poor follow-up.

There was no prior history of central nervous system infections, trauma, or neurocutaneous syndromes. The child had no prior surgical history. He was born full-term via normal vaginal delivery, with a birth weight of 3.1 kg, and no antenatal or perinatal complications. The developmental milestones were normal until 8 years of age, with no prior delays in motor, language, or social development.

Family history was noncontributory, with no known history of epilepsy, neurological illnesses, consanguinity, or metabolic disorders in the family.

Upon examination, the patient was conscious, alert, and oriented to time, place, and person. He responded appropriately to verbal commands and was cooperative during the examination. Vital signs were within normal limits: blood pressure 130/85 mmHg, heart rate 80 beats/min, respiratory rate 18 breaths/min, temperature 98°F, oxygen saturation 98% on room air, with capillary refill time of 2+ bilaterally.

**Fig. 1:**
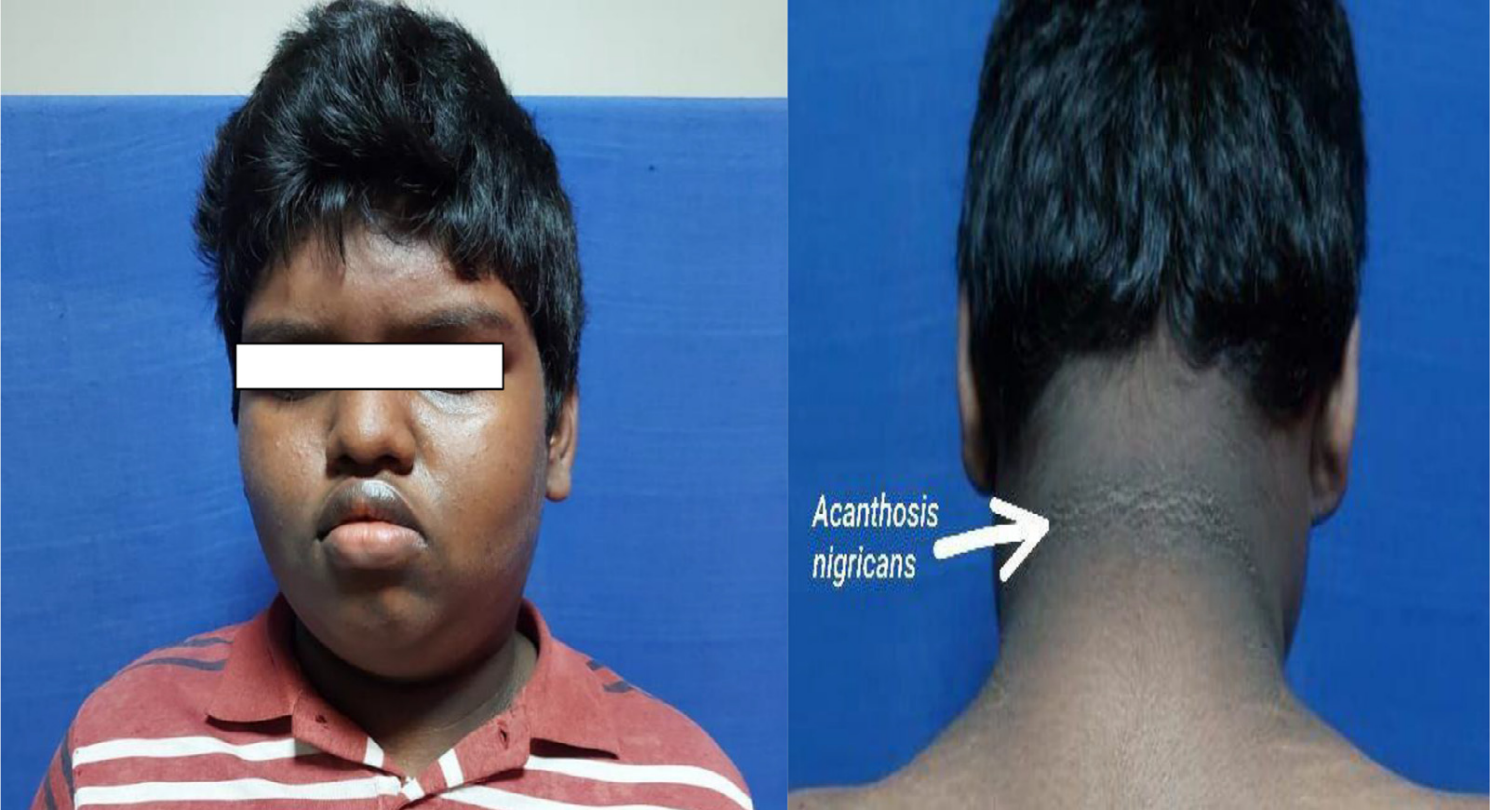
Facial asymmetry and acanthosis nigricans can be seen in the clinical picture.

Anthropometric measurements revealed a BMI of 30.09 kg/m² (obese) and a waist circumference of 112 cm, exceeding age- and sex-adjusted percentiles. Physical examination revealed facial asymmetry (Fig. 1) with flattening on the left side and acanthosis nigricans (Fig. 1) over the neck folds and axillae, suggestive of insulin resistance.

Neurological examination revealed motor deficits on the left side, with muscle power 3/5 in both the upper and lower limbs, accompanied by hypertonia, brisk deep tendon reflexes, and an extensor plantar response. In contrast, the right side demonstrated normal muscle power (5/5), tone, and reflexes, along with a flexor plantar response. Examination of the cranial nerves, cerebellar signs, sensory system, and spine were within normal limits. No signs of meningeal irritation or neck rigidity were observed.

Other systemic examinations, including cardiovascular, respiratory, abdominal, and genitourinary, were within normal limits.

Laboratory investigations revealed that the complete blood count (CBC) was within normal limits, with a white blood cell count of 7800/mm³, hemoglobin of 13.2 g/dL, and platelet count of 245,000/mm³ [5]. Renal function tests showed normal values, including a serum creatinine of 0.8 mg/dL and blood urea nitrogen (BUN) of 12 mg/dL. Liver function tests were also normal, with an ALT of 24 U/L, AST of 28 U/L, and total bilirubin of 0.9 mg/dL. Fasting blood glucose was 106 mg/dL, which is considered borderline elevated (normal: 70-100 mg/dL) [5]. The lipid profile revealed a total cholesterol level of 172 mg/dL (desirable <200 mg/dL), triglycerides at 192 mg/dL (high: >150 mg/dL), HDL at 21 mg/dL (low: <40 mg/dL), and LDL at 112 mg/dL (optimal <160 mg/dL) [5]. Thyroid profile, cortisol levels, and serum insulin levels were all within normal limits.

These findings, particularly obesity, dyslipidaemia, acanthosis nigricans, and elevated waist circumference, met the criteria for metabolic syndrome in a pediatric patient.

**Fig. 2:**
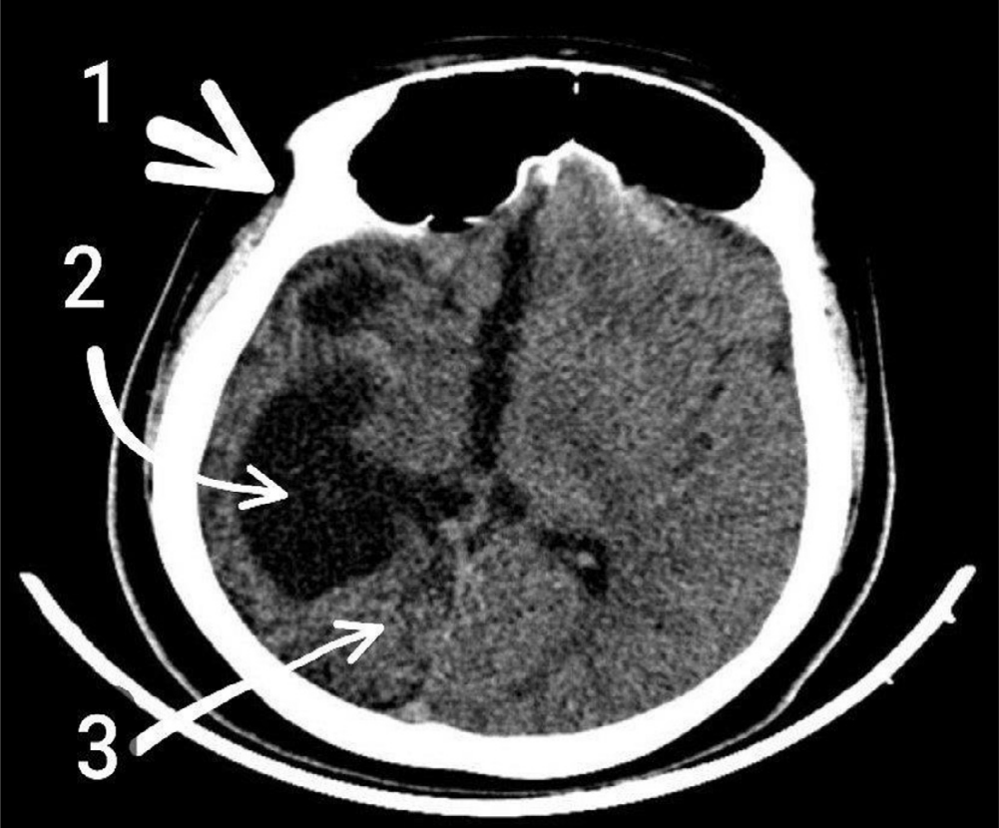
(CT brain—soft tissue window): Noncontrast axial CT scan of the brain in soft tissue window demonstrates marked atrophy of the right cerebral hemisphere with associated ipsilateral ventricular dilatation (ventriculomegaly) and compensatory thickening of the calvarium on the same side. Although optimal visualization of bony structures such as mastoid air cells is limited in the soft tissue window, suggestive features of right-sided mastoid hyperpneumatization can still be appreciated. 1. Calvarial thickening. 2. Ipsilateral ventriculomegaly. 3. Right cerebral hemisphere atrophy.

**Fig. 3:**
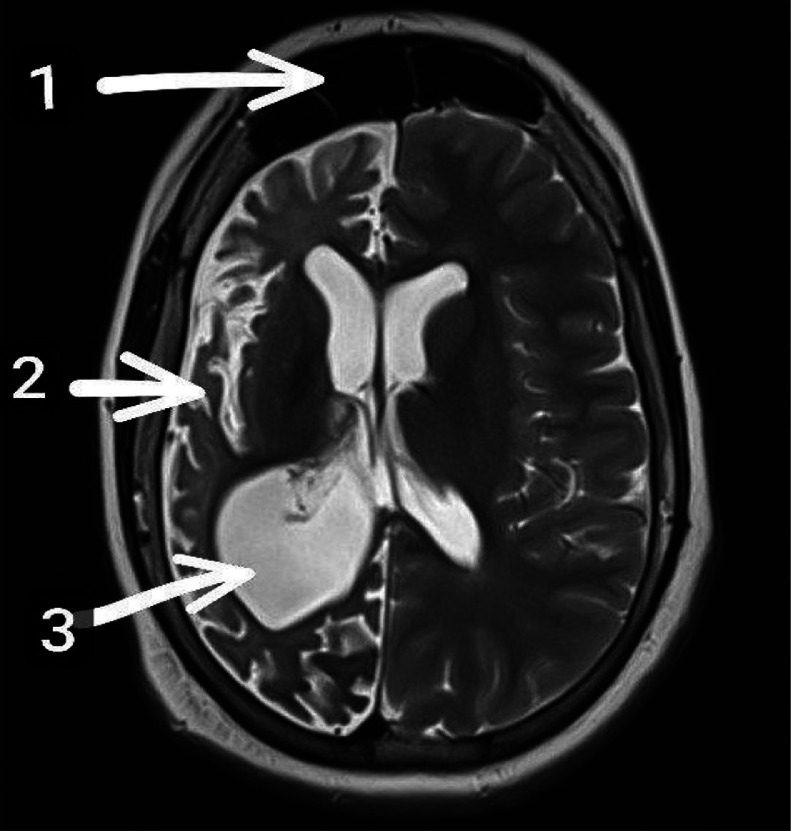
Axial T2-weighted MRI of the brain shows characteristic features of Dyke-Davidoff-Masson syndrome. Note the hyperpneumatization of the right frontal sinus (1), atrophy of the right cerebral hemisphere (2), and ipsilateral (right) ventriculomegaly (3). No contrast-enhanced MRI was performed. 1. Hyperpneumatization of the right frontal sinus. 2. Right cerebral hemisphere atrophy. 3. Ipsilateral (right) ventriculomegaly.

CT scan of the brain revealed classic features of DDMS, including marked atrophy of the right cerebral hemisphere, dilatation of the right lateral ventricle, and calvarial thickening on the right side (Fig. 2). MRI brain further confirmed the diagnosis, showing right cerebral hemiatrophy, ipsilateral (right-sided) ventriculomegaly, and hyperpneumatization of the right frontal sinus, along with a midline shift towards the atrophic hemisphere (Fig. 3). These radiologic features—particularly the combination of cerebral atrophy, compensatory changes in the skull (calvarial thickening and sinus hyperpneumatization), and ventricular enlargement—are characteristic of DDMS.

Based on clinical presentation, history of seizures and hemiparesis, and radiologic findings, a diagnosis of DDMS with coexisting metabolic syndrome was established. The patient was initiated on optimized antiepileptic therapy, including, Levetiracetam 500 mg twice daily, Oxcarbazepine 1800 mg/day in divided doses, Clobazam 10 mg once daily, Topiramate 50 mg twice daily.

He tolerated the medications well with no significant side effects during the inpatient stay. Physiotherapy was initiated for motor rehabilitation, and nutritional counseling was provided to address obesity and dietary habits. The patient and family were counseled on medication adherence, lifestyle modifications, and the importance of regular follow-up. He was discharged in stable condition with outpatient follow-up arranged with pediatric neurology, endocrinology, and physiotherapy services.

## Discussion

Ours is a case of DDMS complicated by metabolic syndrome, characterized by obesity, hypertension, dyslipidemia, and insulin resistance. Imaging was important in differentiating DDMS from other conditions with similar features, such as Rasmussen's encephalitis and Sturge-Weber syndrome (SWS). Symptomatic management focused on antiepileptic medications, alongside lifestyle modifications, including regular exercise, to address the metabolic components. Regular neurological follow-ups were recommended to monitor disease progression and address additional needs such as physiotherapy, to support functional abilities over time.

DDMS is a rare condition, first documented in 1933, and is credited to scientists Masson, Davidoff, and Dyke [6]. The etiology can be congenital or acquired, often due to prenatal or early childhood brain injury [2]. Clinical features of DDMS vary based on the severity of the initial brain insult and can include facial asymmetry, recurrent seizures, contralateral hemiparesis, learning disabilities, intellectual impairment, and delayed developmental milestones [2]. The disease’s natural progression remains poorly understood [7]. Congenital DDMS often presents with vascular abnormalities when it arises during the perinatal period, while the acquired form can develop either perinatally or later, typically due to trauma, infection, or vascular complications [7].

Seizures are the most common clinical manifestation, typically starting early in life as generalized or focal seizures. Patients often require epilepsy management, making DDMS an important differential diagnosis in early-onset epilepsy [8]. In our 13-year-old patient, epilepsy was diagnosed in childhood, but no neuroimaging was conducted—likely due to limited imaging resources at the time. CT and MRI are essential for diagnosing DDMS, with MRI showing characteristic signs such as right cerebral hemisphere atrophy, ventricular enlargement, calvarial thickening, and hyperpneumatization of the right frontal sinus. These findings highlight the importance of advanced imaging in detecting structural abnormalities and differentiating DDMS from other neurological conditions [9]. Notably, this patient also presented with metabolic syndrome, a unique aspect of this case. This association may be due to complications arising from DDMS, such as reduced mobility and hormonal imbalances related to brain injury. A multidisciplinary approach is required, targeting the underlying cause and controlling symptoms [9]. In this case, antiepileptic medications successfully managed generalized tonic-clonic seizures, stabilizing the patient's condition. Due to the rarity of DDMS in infancy and early childhood, individualized treatment plans are necessary, as evidence-based guidelines are still lacking [9].

Differential diagnoses for DDMS include Rasmussen encephalitis, SWS, Silver-Russell syndrome, Fishman syndrome, and hemimegaloencephaly [10]. In SWS, imaging features like ipsilateral skull and sinus changes may mimic DDMS. However, Sturge-Weber typically presents with a facial port-wine stain, pial angiomas, dystrophic cortical calcifications, and an enlarged choroid plexus on the affected side, distinguishing it from DDMS [11]. Rasmussen encephalitis may also appear similar, especially due to its progressive cognitive decline and hemiatrophy involving the frontotemporal, insular, and parietal regions, sometimes with crossed cerebellar atrophy. However, unlike DDMS, Rasmussen encephalitis lacks calvarial changes and predominantly affects children between 6 and 8 years of age, though 10% of cases may present in adolescents or adults [12].

Silver-Russell syndrome (SRS) is an imprinting gene disorder characterized by significant pre and postnatal growth restriction, along with distinctive facial features. These include a triangular face, a pointed chin, micrognathia, a broad forehead, and a thin, wide mouth [2,12]. It may also involve fifth-finger clinodactyly and hemihypertrophy; however, intellectual development generally remains unaffected [2]. Hemimegaloencephaly is characterized by hamartomatous overgrowth of 1 cerebral hemisphere with ipsilateral lateral ventricle enlargement, which may mimic contralateral hemiatrophy [12]. Fishman syndrome, a rare congenital neurocutaneous condition, presents with intellectual disability, ipsilateral cerebral malformations, cerebral calcifications, unilateral temporofrontal lipomatosis, seizures, and leptomeningeal lipomatosis, creating a unique clinical profile [2].

Moreover, emerging literature suggests that chronic neuroinflammation and impaired hypothalamic-pituitary axis function in DDMS may play a pivotal role in the development of metabolic disturbances. These changes, combined with reduced physical activity due to hemiparesis and long-term antiepileptic drug use, can contribute to metabolic syndrome in such patients. While rare, this overlap highlights the need for early metabolic screening and intervention in patients with DDMS to reduce long-term cardiovascular risks. Future studies should focus on explaining the potential pathophysiological mechanisms linking cerebral hemiatrophy and systemic metabolic dysregulation.

## Conclusion

DDMS is a rare neurodevelopmental disorder that remains a diagnostic challenge, particularly in resource-limited settings. Early identification through advanced imaging, especially MRI, is crucial for distinguishing DDMS from other mimicking conditions such as Rasmussen encephalitis and SWS. This case highlights the importance of considering DDMS in pediatric patients with early-onset epilepsy, hemiparesis, and developmental delays. The co-occurrence of metabolic syndrome, though uncommon, may arise due to reduced mobility, long-term antiepileptic therapy, and possible neuroendocrine dysregulation. This unique association highlights the need for a multidisciplinary management approach that integrates neurological, metabolic, and rehabilitative care. Given the absence of standardized treatment protocols, individualized care plans and regular follow-ups are essential. Further research is needed to better understand the long-term outcomes of DDMS and its potential systemic manifestations, including metabolic complications.

## Declaration of generative AI and AI-assisted technologies in the writing process

During the preparation of this work, the author(s) used ChatGPT (OpenAI) in order to improve readability and clarity of the text. After using this tool, the author(s) reviewed and edited the content as needed and take full responsibility for the content of the publication.

## Ethical statement

No ethical approval was required for case reports as per our university guidelines, informed consent from patient/guardian is enough and the same was obtained.

## Availability of data and materials

Not applicable.

## Author contributions

Ramesh Babu S – Idea, conceptualization, supervision, writing draft, approved final draft. Nallathambi – conceptualization, Supervision, writing draft and revision of draft, approved final draft. Shaaruk D– resources, writing draft and revision of draft, approved final draft. Mohammed I – writing draft and revision of draft, approved final draft. Syed N – writing draft and revision of draft, approved final draft.

## Patient consent

Written informed consent for the publication of this case report was obtained from the patient’s parent.
